# Surgical Correction of Thoracolumbar Kyphosis in Achondroplasia: Complications, Pitfalls, and Reflections on the Pursuit of Maximal Realignment in View of Correction Leading to Functional Disability

**DOI:** 10.3390/jcm15083142

**Published:** 2026-04-20

**Authors:** Justyna Walczak, Emilia Nowosławska, Krzysztof Zakrzewski, Paweł Grabala

**Affiliations:** Department of Neurosurgery, Polish-Mother’s Memorial Hospital Research Institute, Rzgowska 281/289, 93-338 Lodz, Poland; justyna.walczak@iczmp.edu.pl (J.W.); emilia.nowoslawska@iczmp.edu.pl (E.N.); krzysztof.zakrzewski@iczmp.edu.pl (K.Z.)

**Keywords:** posterior spinal fusion, achondroplasia, surgical complications, congenital kyphosis, vertebral column resection, VCR

## Abstract

**Background:** Achondroplasia, the most common genetic dwarfism caused by the FGFR3 mutation (autosomal dominant, 80% de novo), results in a disproportionately short stature. Thoracolumbar kyphosis (TLK), combined with characteristic spinal canal stenosis, increases the risk of symptomatic compression, yet the literature lacks clear thresholds for symptom onset or progressive deformity angles. **Methods:** A 16-year-old female with achondroplasia presented with rapidly progressive kyphosis despite conservative management (bracing and therapy). Over six months, she developed neurogenic claudication; bilateral leg pain; weakness; and paresthesia that worsened with standing/walking, which was relieved by flexion/sitting. Imaging demonstrated surgical-threshold kyphosis with progressive spinal misalignment. Her symptoms indicated compressive myeloradiculopathy from lumbar stenosis, critical given achondroplasia’s congenitally narrowed canal and heightened neurologic vulnerability. **Results:** Staged surgery planned: Posterior fusion T6-L4 with pedicle screws and then extensive decompression (laminectomy/foraminotomy T11-L3), L1 corpectomy with expandable titanium cage, and Ponte osteotomies. Intraoperative complications included a malpositioned left T10 screw breaching the anterior/lateral cortex near the aorta, requiring urgent revision. Postoperatively: Neurogenic bladder, wound leakage, and *E. coli* urinary tract infection (UTI) with fever (treated with IV antibiotics). After infection resolution, definitive surgery removed the malpositioned screw and completed decompression, corpectomy, cage placement, bone grafting, and osteotomies, successfully resolving neurological symptoms. However, 13 cm trunk lengthening caused severe functional impairment—disproportionately short arms prevented independent toileting and dressing. Left arm lengthening via external fixation restored partial function. At 2.5-year follow-up, there was solid fusion, no neurological deficits, and improved quality of life. **Conclusions:** Surgery addresses severe TLK, vertebral wedging, and neurogenic claudication in achondroplasia. Vertebral column resection effectively corrects TLK and neurological deficits but carries a high complication risk. This should be reserved for severe TLK with hypoplastic vertebrae, performed by experienced surgeons. Critically, correction magnitude must preserve limb–trunk proportions to prevent functional disability, as excessive lengthening may necessitate additional limb procedures for independence restoration.

## 1. Introduction

Achondroplasia is a type of skeletal dysplasia that results in a disproportion of height [1,2] and limb length. Parts of arms and legs are shortened; moreover, the shapes of the head and spine are often altered [3,4,5,6,7,8]. This occurs due to a gain-of-function mutation in the fibroblast growth factor receptor 3 (FGFR3) gene [9,10]. The growth factor receptor 3 (FGFR3) gene normally slows the growth of bone that forms from cartilage. This mutation causes the fibroblast growth factor receptor 3 (FGFR3) gene to be expressed excessively [10,11]. The mutation results in a suppression of the transformation of cartilage into bone. It impairs the growth of bones and vertebrae. The incidence is one in fifteen thousand to one in twenty-five thousand births, although the actual prevalence may be marginally elevated [11,12]. It is commonly diagnosed as the genetic cause of dwarfism. De novo mutations are responsible for approximately 80% of diagnosed cases of achondroplasia [13,14]. Similar to numerous autosomal dominant disorders, a positive correlation exists between advanced paternal age and the incidence of novel mutations [15]. Anomalies in achondroplasia include a shortening of vertebral pedicles in the thoracolumbar region and a narrowing of interpedicular distance in the lumbar spine [3,16,17,18]. Skeletal anomalies in achondroplasia lead to an increase in spinal canal stenosis and craniovertebral junction stenosis with cervicomedullary compression [19,20,21,22]. Spinal canal stenosis can manifest as symptoms such as claudication, radiculopathy, or severe cases of myelopathy. The cranial pathology associated with achondroplasia is further intensified by the reduced dimensions of the posterior cranial fossa, which arises from the inhibited growth of the nuchal plane, which is a product of endochondral ossification, in conjunction with the secondary horizontalization of the squamous segments of the occipital bone [9,23,24,25]. This “congested” anatomy of the skull base results in the displacement of the brainstem in a superior direction and the foramen magnum in an anterior direction, thereby inducing a posterior angulation of the foramen magnum and exacerbating the posterior compression of the neuraxis [5,13,25,26,27]. Sometimes procedures such as foramen magnum decompression or lumbar canal decompression need to be performed to relieve nerve compression in symptomatic patients [28,29]. Skeletal dysplasias other than achondroplasia show a high incidence of spinal deformities such as scoliosis and kyphosis. In clinical practice, it is rather common to encounter patients with achondroplasia who develop neurologic deficits due to sagittal spinal malalignment [17,30,31]; corrective surgeries for such patients may be considered. The management of cervicomedullary compression necessitates a prompt and assertive intervention. Empirical evidence indicates that this condition is progressive and potentially lethal, as it heightens the likelihood of sudden death resulting from central respiratory failure [4,12,32,33]. This pathology is increasingly acknowledged as a contributing factor to respiratory and neurological anomalies in pediatric patients diagnosed with achondroplasia [12,34,35,36]. Pedicle subtraction osteotomy (PSO) is a spinal reconstructive technique, as is vertebral column resection (VCR); the latter, however, is associated with greater risk for the patient [37,38]. Pedicle subtraction osteotomy (PSO) and vertebral column resection (VCR) present a potential for realignment in the sagittal plane. Reports of interventions in cases of patients with achondroplasia are rare, and long-term outcomes remain poorly documented.

In this paper, we present a technical note on the surgical management of a 16-year-old girl with achondroplasia with severe congenital kyphosis and complications in surgical management with a 2-year follow-up. This case provides a rare insight into the long-term effectiveness, safety, and alignment outcomes of corrective osteotomy in achondroplastic individuals with complex spinal deformities.

## 2. Materials and Methods

### 2.1. Study Design

This study was designed as a retrospective review and illustrative surgical technique report. The primary aim was to describe complications, pitfalls, and reflections on the pursuit of maximal realignment, with illustrative models and a representative clinical case. Pedicle screw fixation was achieved using the Reline^®^ spinal system (Globus Medical, Inc., Audubon, PA, USA).

### 2.2. Illustrative Model

**Figure 1:**
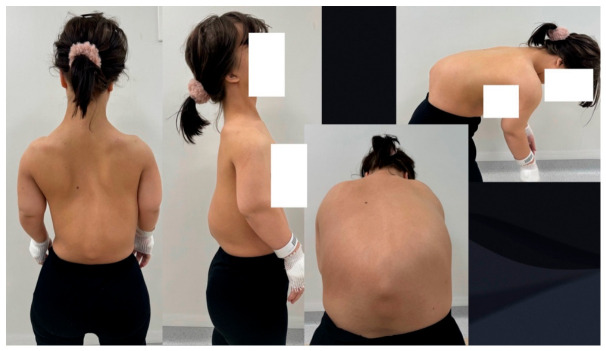
Clinical pictures show a 16-year-old girl with achondroplasia and congenital severe kyphosis before surgical treatment.

A 16-year-old female patient with achondroplasia presented to an orthopedic spine clinic due to a rapidly increasing sagittal plane spinal deformity in the lumbar region (Figure 1).

She had earlier received non-surgical care at a different health center, which included a set plan with spinal bracing and supervised therapy. Subsequent scans of her spine showed a progressive deterioration of the spinal alignment. The Kyphotic Cobb angle reached the point at which surgical intervention is often considered. In the six months prior to her visit to the orthopedic clinic, the patient’s condition worsened. She began to experience signs of neurogenic claudication and felt pain in both legs, which increased over time; at the time of admission, she had stopped going to school and was unable to freely walk around her house. She also reported muscle weakness and tingling sensations, which were aggravated by standing upright or walking and slightly relieved by bending forward or sitting down. This description matches the clinical manifestation of lumbar spinal stenosis. Given the patient’s prior diagnosis of achondroplasia—a skeletal dysplasia characterized by congenital narrowing of the spinal canal and increased susceptibility to progressive kyphotic deformity—the symptom complex strongly indicated compressive myeloradiculopathy secondary to spinal stenosis. This diagnosis is particularly concerning in patients with achondroplasia, where compromised canal diameter and altered vertebral morphology substantially heighten the risk of early and severe neurologic impairment [4,17,39].

Clinical assessment demonstrated typical signs of achondroplasia. The patient displayed classic phenotypic features of achondroplasia, including disproportionate short stature with characteristic rhizomelic limb shortening, macrocephaly with prominent frontal bossing, and midface hypoplasia (Figure 1). Spinal examination revealed severe thoracolumbar kyphosis measuring more than 100 degrees, accompanied by compensatory exaggerated lumbar lordosis. She demonstrated a characteristic forward-leaning trunk posture, indicating significant disruption of normal sagittal spinal balance and compensatory mechanisms.

Comprehensive neurological assessment revealed concerning signs of spinal cord dysfunction, including hyperreflexia in the lower extremities, bilateral positive Babinski signs, and mild but definite weakness in L2–L4 myotomal distributions. Sensory testing demonstrated diminished light touch and vibratory sensation in the L1–L3 dermatomes bilaterally. Functional walking assessment provoked characteristic symptoms of pain and formication, which improved significantly after lumbar spine flexion‚ confirming the diagnosis of neurogenic claudication secondary to spinal stenosis. Range of motion testing revealed severe limitations in spinal extension and lateral bending, with compensatory hip flexion contractures. Pulmonary function remained within normal limits, even though patients with achondroplasia frequently demonstrate restrictive respiratory patterns due to thoracic cage abnormalities and reduced chest wall compliance.

**Figure 2:**
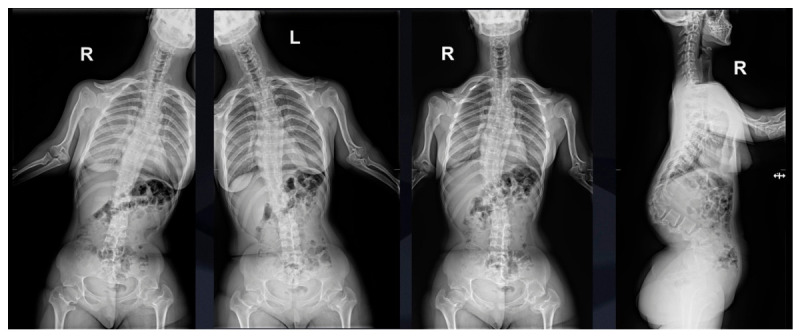
X-ray AP and lateral and bending films of a 16-year-old girl with achondroplasia and congenital severe kyphosis before surgical treatment.

Standing full-spine radiographs of both anteroposterior and lateral projections confirmed severe thoracolumbar kyphosis measuring more than 100 degrees on the Cobb scale (Figure 2), accompanied by a secondary coronal plane curvature. The L1 vertebra exhibited classic signs of dysplastic wedging, including significant anterior height loss and abnormal posterior element development.

**Figure 3:**
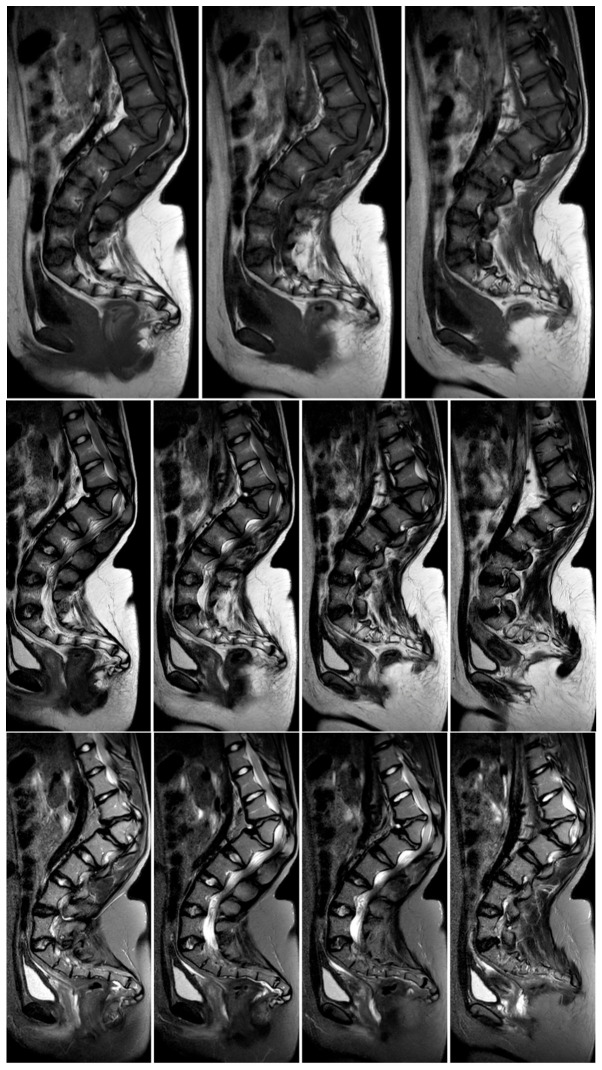

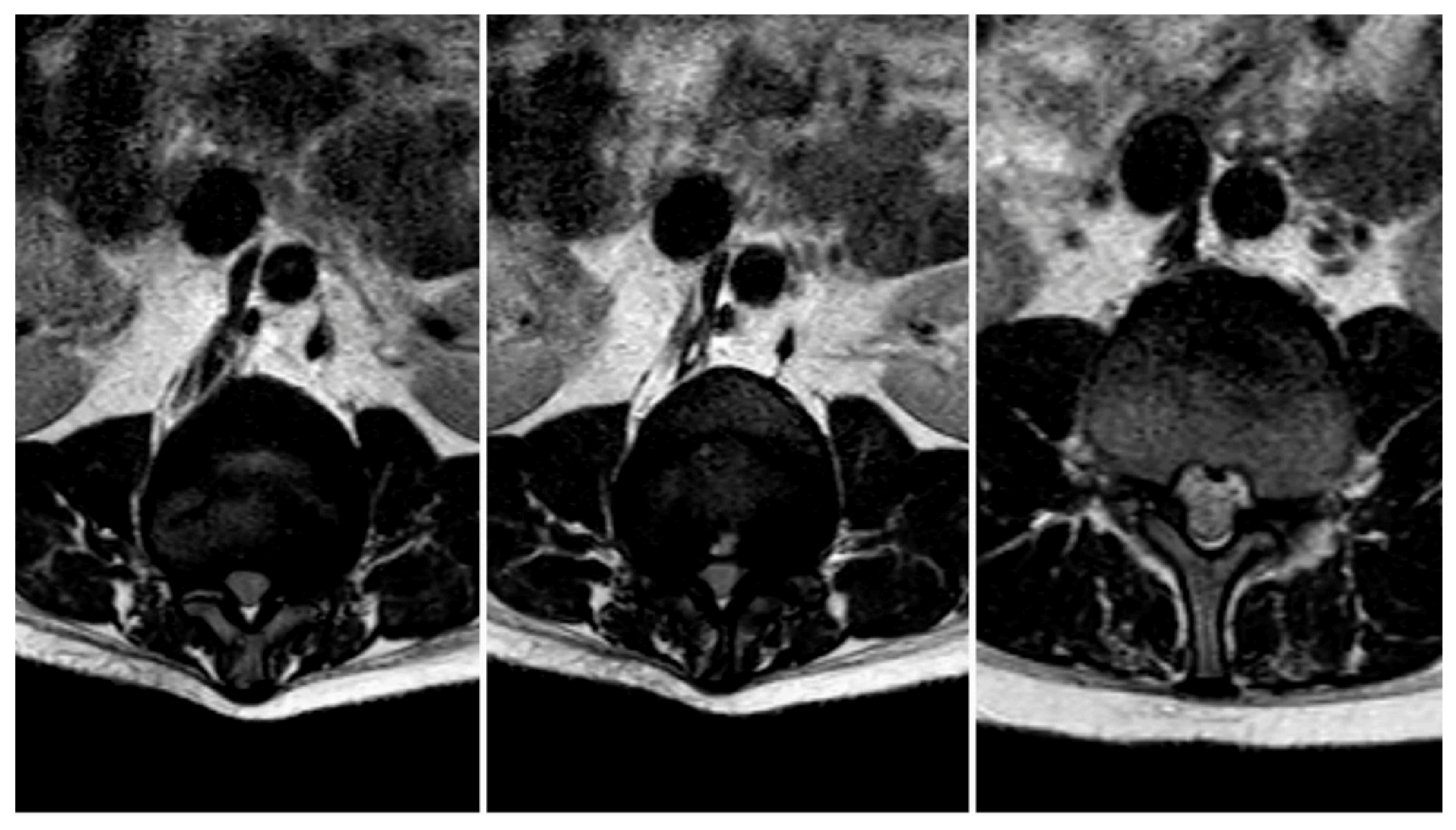
MRI of a 16-year-old girl with achondroplasia and congenital severe kyphosis before surgical treatment.

Comprehensive high-resolution imaging, including X-rays, magnetic resonance imaging and computed tomography with multiplanar reconstruction, revealed severe dysplastic changes at the L1 vertebral level, with significant structural abnormalities contributing to both progressive spinal deformity and neural compression (Figure 3).

**Figure 4:**
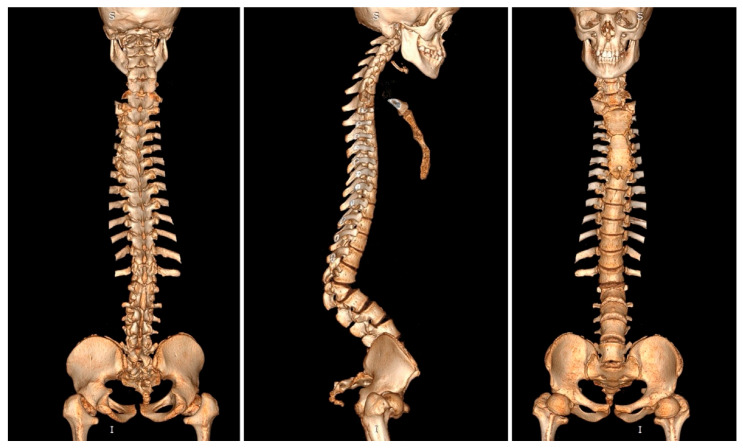
Preoperative 3D-CT of a 16-year-old girl with achondroplasia and congenital severe kyphosis.

The spinal cord demonstrated significant compression at the thoracolumbar junction, which correlated directly with the patient’s neurological symptoms. The conus medullaris appeared deformed and compressed, with additional multilevel spinal stenosis extending from T11 to L3 involving both the central canal and bilateral neural foraminal narrowing. No fluid reserve was visible at the level of compression; however, there was no sign of myelopathy when the imaging was being done. Three-dimensional computed tomography reconstruction provided exquisite detail of the dysplastic vertebral anatomy, enabling precise surgical planning and identification of potential anatomical challenges (Figure 4). The comprehensive imaging findings confirmed the complexity of the case and established the absolute necessity for major surgical intervention to prevent permanent neurological deterioration.

### 2.3. Surgical Planning and Management

The initial surgical approach involved performing a two-stage surgery. Firstly, posterior spinal fusion utilizing pedicle screw instrumentation from T6 to L4 was designed to achieve spinal stabilization and deformity correction through carefully planned distraction and compression maneuvers applied through a posterior instrumentation system. In the second stage, the surgical procedure consisted of multiple additional components: extensive spinal cord decompression (laminectomy and foraminotomy from T11 to L3) to achieve adequate neural decompression, careful egg-shell osteotomy with subtotal corpectomy of the dysplastic L1 vertebra, complete discectomy at the T12-L1 level, and placement of an expandable titanium mesh cage prosthesis at L1 to restore anterior column support. Structural autologous bone grafting was to be utilized to enhance anterior spinal support and facilitate solid arthrodesis. Multiple Ponte osteotomies were planned at various levels to achieve optimal spinal realignment and maximize neural decompression. These comprehensive surgical techniques were intended to allow for substantial correction of the severe spinal deformity while directly addressing the underlying pathophysiology responsible for the patient’s progressive neurological symptoms.

## 3. Results

**Figure 5:**
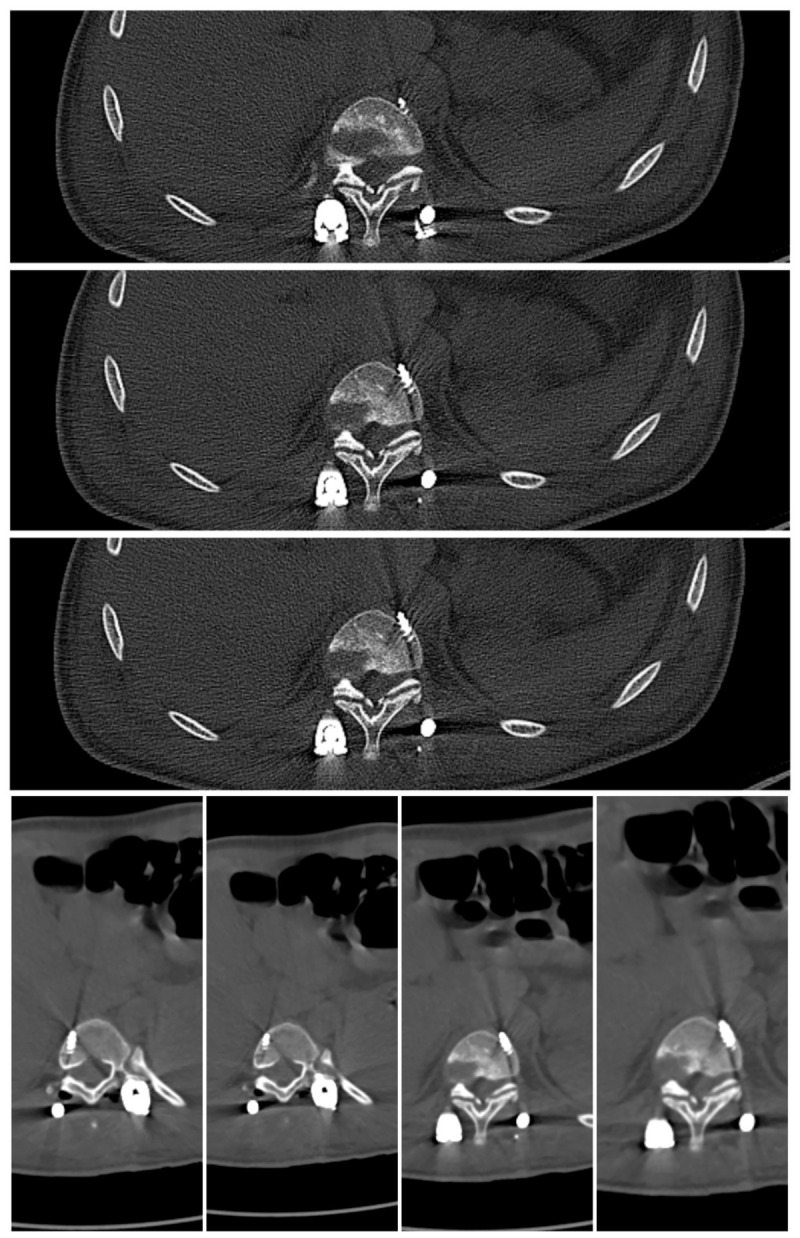
CT at the T10 level in a patient with achondroplasia showing the characteristic congenitally narrow spinal canal and dysplastic, narrowed pedicles. A left-sided transpedicular screw traverses the pedicle with anterior and lateral cortical breach of the vertebral body; the screw tip projects ~5 mm beyond the cortex, in close proximity to the thoracic aorta. The screw was removed during revision surgery.

**Figure 6:**
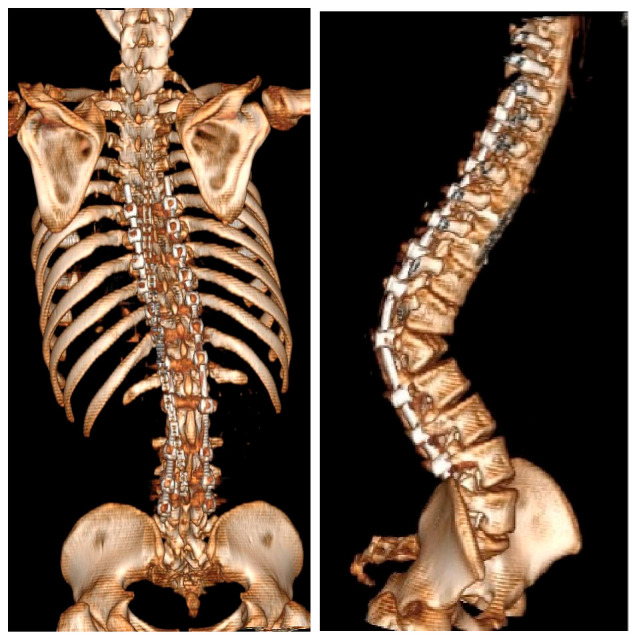
3D-CT after the first stage of planned surgical treatment.

As unforeseen difficulties occurred, the preoperative surgical plan required modification. Significant intraoperative complications occurred during the procedure. A pedicle screw incorrectly positioned at the T10 level required immediate revision in order to prevent catastrophic vascular injury; through postoperative CT scanning, it was discovered that the left T10 pedicle screw had breached the anterior and lateral cortex and was positioned dangerously close to the descending thoracic aorta, creating an imminent risk of life-threatening vascular injury (Figure 5 and Figure 6).

**Figure 7:**
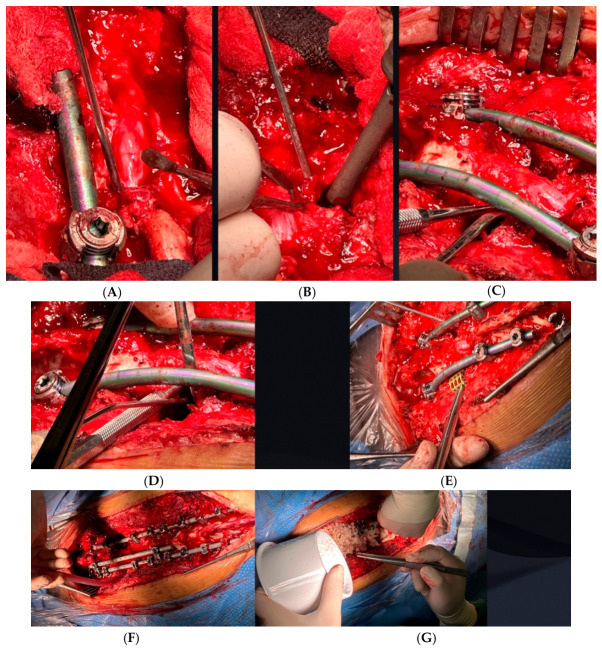
Intraoperative picture of Vertebral Column Resection of L1 and T6-L4 PSF. (**A**) Spinal cord decompression of L1. (**B**) Egg-shell subtotal corpectomy of L1, manual sideways shifting of the dural sac. (**C**) L1 body removed. (**D**) Manual vertical shifting of the dural sac and posterior wall removal, L1 vertebral body removal visible. (**E**) Vertebral body cage implantation at L1. (**F**) Fixation system with 3 stabilizing rods. (**G**) Bone graft implantation.

Moreover, on the fourth day post-op, neurological bladder was observed, and fluid leakage from the wound occurred. A bacterial urinary tract infection (*E. coli*) with fever was diagnosed. Intravenous antibiotic therapy was initiated immediately; only after the inflammation marker levels decreased was the final surgery performed. Following detailed analysis of the postoperative CT findings, the malpositioned T10 screw was carefully removed to eliminate the risk of aortic injury while maintaining adequate fixation strength. The surgical procedure was expanded to include multiple additional components: extensive laminectomy and foraminotomy from T11 to L3 to achieve adequate neural decompression, careful egg-shell osteotomy with subtotal corpectomy of the dysplastic L1 vertebra, complete discectomy at the T12-L1 level, and expandable titanium mesh cage prosthesis placement at L1 to restore anterior column support. Structural autologous bone grafting was utilized to enhance anterior spinal support and facilitate solid arthrodesis. Multiple Ponte osteotomies were performed at various levels to achieve optimal spinal realignment and maximize neural decompression. These comprehensive surgical techniques allowed for substantial correction of the severe spinal deformity while directly addressing the underlying pathophysiology responsible for the patient’s progressive neurological symptoms (Figure 7).

**Figure 8:**
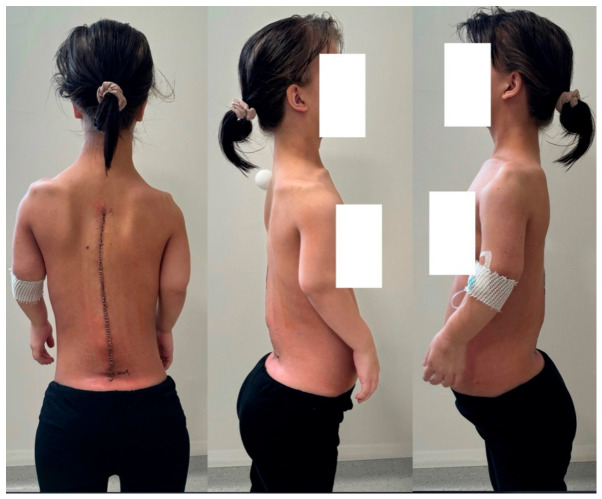
Postoperative clinical pictures after surgical treatment.

**Figure 9:**
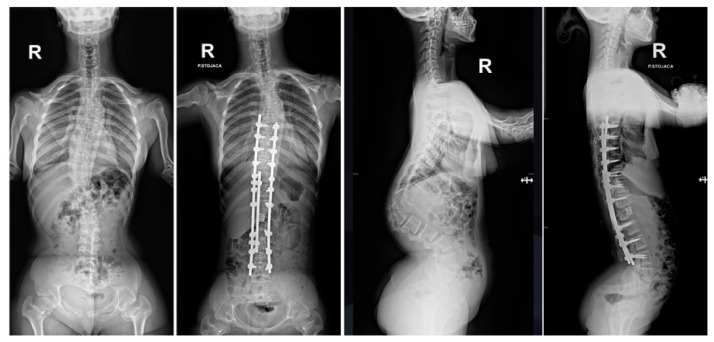
Postoperative X-rays after surgical treatment in comparison with pre-operative X-rays.

Excellent results were achieved through the surgical intervention in resolving primary neurological complications. The patient’s neurogenic claudication symptoms resolved completely, and normal bladder function was restored (Figure 8 and Figure 9). The drastic correction resulted in an approximately 13 cm trunk height gain.

**Figure 10:**
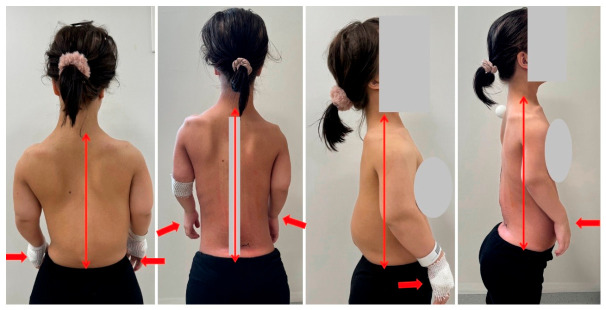
The image presents a comparison of body proportion changes following spinal deformity correction. The arrows indicate how the proportions of the upper limbs relative to the trunk have changed.

Immediately following the surgery, the patient expressed significant dissatisfaction with the outcome and the effects of the intervention. Her prior symptoms of neurogenic claudication and bladder dysfunction appeared to be overshadowed or forgotten in light of her new postoperative complaints. She reported a noticeable decline in her overall quality of life. Following the surgery, her trunk length increased by approximately 13 cm. Her upper limbs became disproportionately short relative to her torso. She was unable to independently put on her underwear or trousers. She was no longer able to clean herself after defecation without assistance. She had completely lost the ability to manage her personal hygiene in that regard. Overall, she became functionally dependent and unable to care for herself independently. Figure 10 demonstrates the extent of the problem and the associated functional impairment.

**Figure 11:**
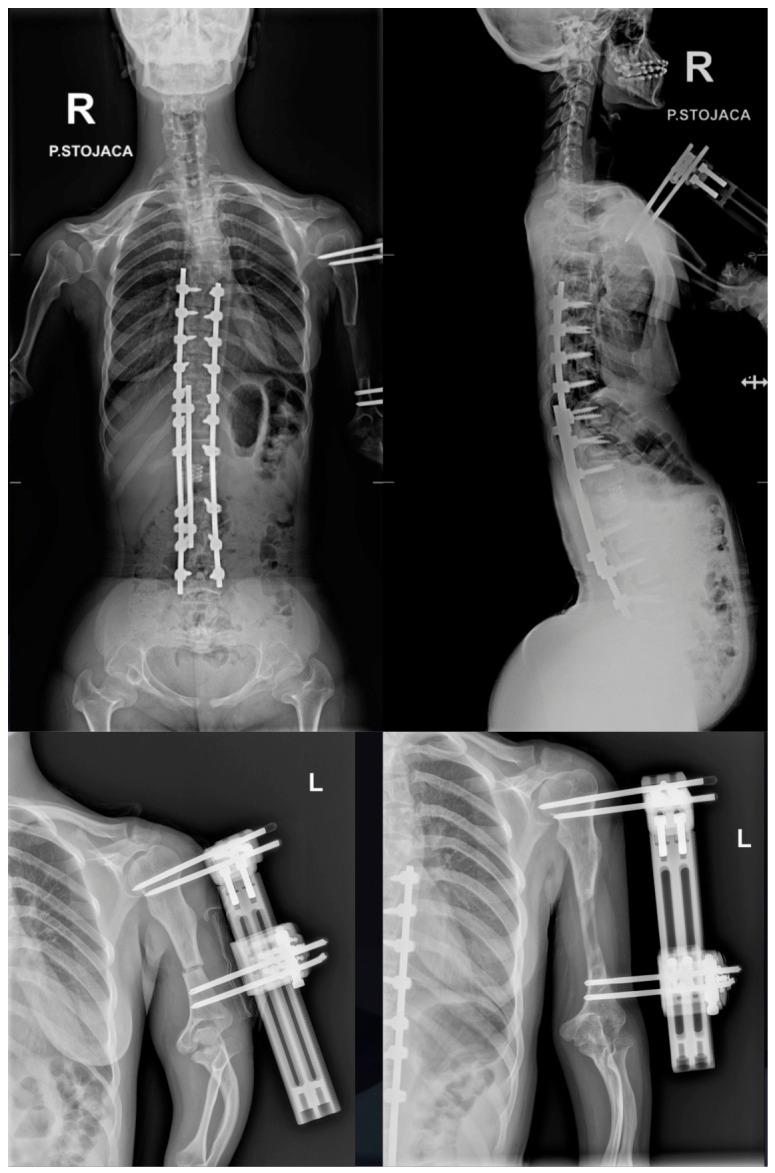
Radiographic images showing the left upper limb during the distraction phase of the lengthening procedure.

We consulted a colleague from the Department of Orthopedics to consider surgical limb lengthening due to the patient’s disproportionate body proportions. An arm-lengthening procedure was shortly performed thereafter using a monolateral external fixation frame. The patient’s left upper limb has been lengthened by 6 cm. For now, she has chosen not to proceed with lengthening the right upper limb (as shown in Figure 11).

**Figure 12:**
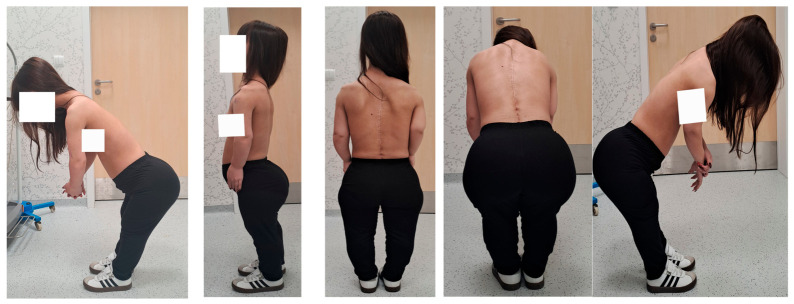
Clinical pictures show a 16-year-old girl with achondroplasia and congenital severe kyphosis after undergoing surgical treatment at the most recent follow-up and clinical evaluation (2.5 years after surgery).

**Figure 13:**
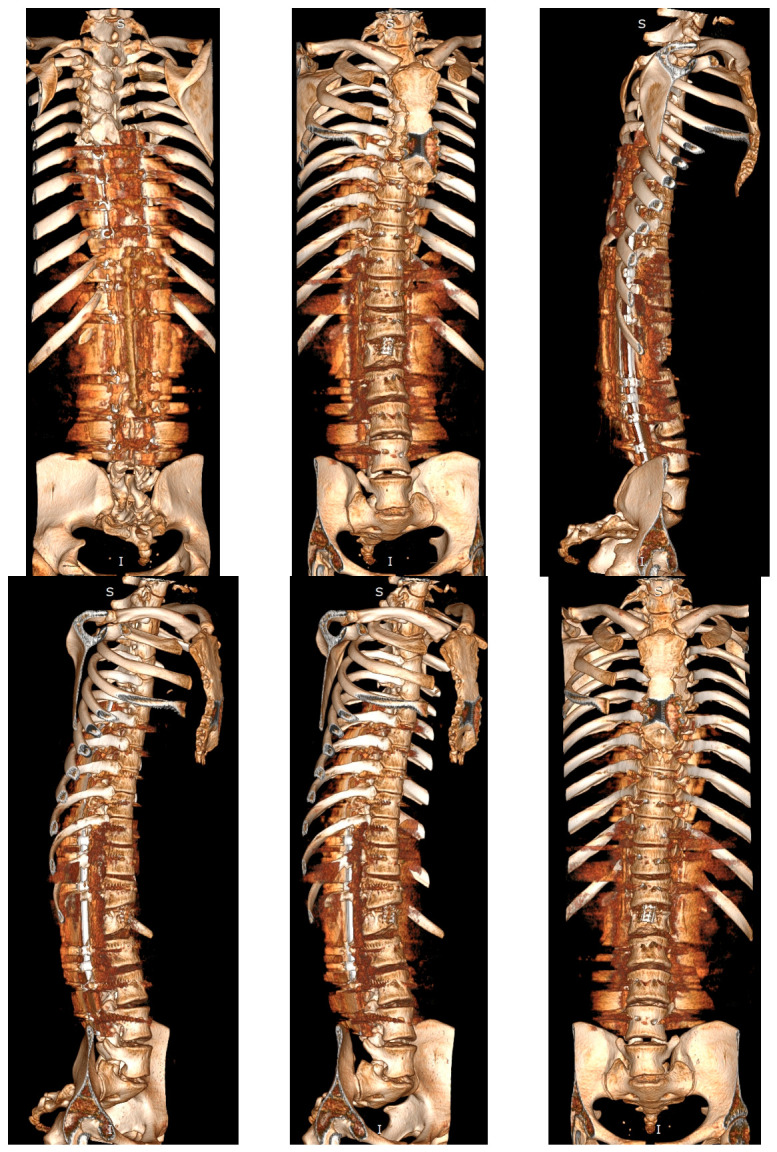
Postoperative 3D-CT of a 16-year-old girl with achondroplasia and congenital severe kyphosis after undergoing surgical treatment at the most recent follow-up and clinical evaluation (2.5 years after surgery).

At the most recent follow-up and clinical evaluation (2.5 years after surgery), the patient reported no complaints and demonstrated no neurological deficits. She was not taking any medications and reported feeling well. However, an overall improvement in her quality of life was hindered by the fact that the limb lengthening procedure was unsuccessful in terms of her regaining independence. Her left arm was lengthened insufficiently for her to be able to manage her own hygiene. Follow-up imaging, including clinical pictures and computed tomography, confirmed solid spinal fusion at the operated levels and successful spondylodesis within the instrumented segments (Figure 12 and Figure 13).

## 4. Discussion

Achondroplasia is classified within the category of skeletal dysplasia and arises from aberrant endochondral ossification [1,2,3,13]. A positive correlation exists between advanced paternal age and the incidence of novel mutations [9,10,15,40]. Clinically, achondroplasia is characterized by disproportionate short stature accompanied by rhizomelic limb shortening, macrocephaly, midfacial hypoplasia, and frontal bossing [3,41,42]. The morbidity associated with achondroplasia is primarily due to osseous compression of the neuraxis [13,19] and respiratory insufficiency [23,25]. Spinal and neurological disorders that may necessitate surgical intervention include cervicomedullary compression and/or instability, spinal canal stenosis, and thoracolumbar hyperkyphosis resulting from congenital vertebral malformations [26,40,43]. In all cases of symptomatic disorders such as these, an early surgical decompression is advised; compared to late interventions, early surgery is more likely to improve neurological symptoms [44]. The majority of individuals with achondroplasia demonstrate normal cognitive abilities; however, psychosocial challenges related to reduced stature are prevalent, commonly manifesting as peer rejection and a propensity for adults to regard individuals with achondroplasia based on their height rather than their chronological age [1,3]. The heightened mortality rate in childhood is likely linked to severe cervicomedullary compression; thus, a heightened awareness of potential health complications associated with achondroplasia, along with appropriate diagnostic evaluations and targeted interventions, is imperative [1,16,25,41]. Thoracolumbar spinal canal stenosis resulting in spinal cord compression is the most prevalent pathological condition observed in patients with achondroplasia, typically becoming symptomatic in the majority of cases during their third decade of life or after [12,45,46,47]. Of congenital spinal anomalies that occur in these cases, progressive thoracolumbar kyphosis is the most common in patients with achondroplasia. Thoracolumbar stenosis may significantly worsen during infancy if brace treatment is not commenced prior to the onset of vertebral wedging [39,42,48] or if surgical intervention is postponed. Less prevalent issues in infancy may include symptomatic upper airway obstruction [3,16,25,43] and severe cervicomedullary compression secondary to foramen magnum stenosis [25]. The latter may present with dysphagia and central sleep apnea [27].

The anatomical structure of the spine in patients with achondroplasia shows distinctive characteristics that predispose to compression of both the spinal cord and nerve roots [30,49]. Hypertrophy observed in the metaphyseal articular surfaces of long bones is paralleled by similar changes at the cranial and caudal endplates of the vertebral bodies, imparting a “mushroom-like” morphology at both extremities and leading to concomitant posterior scalloping detectable via contrast myelography [1,13]. The occurrence of shortened and thickened pedicles is attributed to the premature fusion of the synchondroses that exist between the neural arches and the vertebral bodies [13,50]; additionally, the laminae themselves also exhibit thickening. Intervertebral discs frequently present with significant bulging [18,19,21], which further amplifies neural compression resulting from the enlarged articular surfaces of the vertebral bodies. The interpedicular distance diminishes in the caudal segment of the lumbar spine, thereby inducing a progressive constriction of the spinal canal in the caudal direction [21,23], in contrast to the normal state wherein the canal typically expands in the caudal end. Together, these anatomical features result in pronounced multiaxial stenosis of the spinal canal, which may be exacerbated by degenerative changes and annular fissures associated with the intervertebral discs [20,23]. Neurological symptoms manifesting below the foramen magnum generally arise in late adolescence and adulthood, most likely as a consequence of postural or degenerative alterations superimposed upon pre-existing congenital stenosis. Research studies indicate that 35% of patients exhibit symptomatic manifestations prior to reaching the age of 15 years [12,42,45,51]. Estimates regarding the prevalence of symptomatic stenosis fluctuate between 37.5% and 89%, suggesting that a significant proportion of patients are destined to develop this condition over time [12,42,45]. Given that the spinal canal in achondroplasia may be congenitally narrow, enhanced early screening protocols could facilitate the identification of a considerable number of young patients exhibiting subclinical signs of stenosis [13,17]. While symptomatic stenosis may necessitate neurosurgical intervention, the neurosurgical and orthopedic management facets of the achondroplastic spine can typically be delineated, as certain neurological complaints warranting surgical intervention are secondary to evolving orthopedic issues [17,20,50]. The postural positions of sitting and standing have a significant impact on spinal curvatures, and in pediatric patients with achondroplasia, these biomechanical implications are further exacerbated by factors such as muscular weakness, shorter pedicles, and ligamentous laxity in the spine [9,23]. The dynamic repercussions of a diminished thoracic cage combined with an anteriorly protruding abdomen on the progression of kyphosis have been notably emphasized in scholarly discourse [40,52]. Furthermore, a delayed initiation of standing activities predisposes patients to the formation of a gibbus characterized by the wedging of one or multiple vertebral components. Such deformities are debilitating; however, they are also recognized as subject to preventive measures. Considering complications associated with surgical intervention, it is prudent to prioritize preventive strategies [24,43,49]. The application of prophylactic orthotic bracing may be advisable or, according to some professionals, even necessary, in scenarios where the likelihood of wedge-shaped gibbus formation is anticipated [53], although it will not prevent trunk–limb disproportion.

In adult populations, spinal compression may arise from various abnormalities, including hyperlordosis, mild disc protrusions, degenerative changes of a hypertrophic nature, or ligamentous hypertrophy [25,40,41]. The occurrence of thoracolumbar kyphosis exhibits a positive correlation with symptomatic stenosis [4,45]. While low back pain is frequently reported by patients with achondroplasia, those experiencing significant stenosis may develop symptomatic neurogenic claudication. Extended periods of ambulation typically elicit paresthesia, which is subsequently followed by muscular weakness in the lower extremities, generally bilaterally. These symptoms are promptly alleviated through rest, squatting, or forward flexion, all of which serve to diminish lumbar lordosis and increase the transverse diameter of the lumbosacral canal [39,42,54]. As stenosis advances, the distance a patient can walk before the onset of claudication diminishes, rendering this metric clinically significant.

In cases of advanced stenosis, neurological deficits, such as weakness in the lower extremities (notably affecting toe and ankle dorsiflexors) and hypoesthesia, often at the level of the trunk, may persist even during periods of rest [39,42]. Instances of partial Brown–Séquard syndrome may be observed sporadically. Spasticity and hyperreflexia in the lower extremities typically indicate compression of the thoracic spinal cord but may also imply concurrent cervical compression [45,46]. In addition to these observations, the neurological examination of patients presenting with claudication is frequently otherwise unremarkable, with the exception of the existence of concomitant disc pathology. It is imperative that the thoracolumbar spine be assessed in all symptomatic patients with achondroplasia, even when overt neurological signs are absent. The manifestation of urinary incontinence or difficulties with voiding should be regarded as an urgent cue for diagnostic investigation. The indications for surgical intervention in cases of spinal canal stenosis are contingent upon the clinical presentation [13,17,18,31,50].

The clinical case presented and analyzed by us demonstrates that the patient developed progressive narrowing of an already congenitally narrow spinal canal (which is anatomically reduced in achondroplasia). Due to a congenital spinal anomaly, namely, a wedge vertebra at the thoracolumbar junction, the patient experienced a gradual, slow progression of spinal canal narrowing secondary to a kyphotic spinal alignment. Approximately 25% of patients with achondroplasia develop spinal canal stenosis [55], and the primary treatment modality is neural decompression via laminectomy [28]. However, decompression performed without transpedicular stabilization leads to progressive instability in up to 17% of cases [55] and, in some instances, to the development of secondary spinal deformities, such as post-laminectomy thoracolumbar kyphosis [28]. Hallan et al. recommend preoperative planning for extension of the surgical procedure in cases requiring multilevel laminectomies and consideration of stabilization already during the initial operation [56]. In the case of our patient, thoracolumbar kyphosis was not related to a previous surgery; she was most likely among the 10% of cases of patients in which kyphosis did not resolve at 18 months of age, which is the average age of walking [57]. For such patients, surgical correction for thoracolumbar kyphosis should be considered whenever the kyphotic Cobb angle is calculated to be above 50 degrees [55]. Yilar et al. [58], in one such case, performed a segmental instrumentation with a sliding growing rod system, successfully decreasing the kyphotic Cobb angle from 45 to 11.3 degrees. However, due to our patient’s age, we did not find it necessary for the system to be extended and therefore implanted a traditional non-growing stabilization system. The severe spinal deformity was corrected, as seen in the attached images of pre- and postoperative X-rays and CTs, and sagittal plane malalignment was effectively improved. In [45], the authors suggest that not only should new neurological deficits cause the operator to consider whether or not a screw system is suboptimally positioned but also a lack of neurological improvement, i.e., neurogenic claudication, weakness, numbness, reflex incontinence and so forth; persistence might indicate not only the wrong level being operated but also an incorrect position of the stabilizing system [56]. Decompressive laminectomy is regarded as the primary intervention for individuals diagnosed with achondroplasia who experience claudication that markedly restricts ambulation, causes significant weakness at rest (excluding minor weakness of the extensor hallucis longus), or causes urinary incontinence/urgency resulting from compression of the cauda equina or spinal cord. It is crucial to emphasize that urological complications frequently arise post-laminectomy in patients with achondroplasia, and there is a significant association between preoperative urological dysfunction and subsequent postoperative urinary issues [59], as occurred in the presented case. Special consideration should be directed towards manifestations of neurogenic bladder, which may emerge either prior to surgery or as a result of decompressive procedures [9,10,23,26]. Consequently, a thorough urological assessment is advised as an integral component of the preoperative evaluation. The process of decompressing the spinal canal in individuals with achondroplasia presents significant technical challenges due to the magnitude and severity of stenosis [24,26,49]. Historically, reports of unsatisfactory postoperative results ensuing from decompression in achondroplastic patients have been relatively prevalent [1,30]. The utilization of bulky instruments beneath the laminae in conventional methodologies has frequently led to injury of neural tissue. Additionally, another contributing factor to poor outcomes has been the postoperative instability resulting from excessively extensive laminectomies [13,16,30]. An alternative strategy to achieve an optimal laminectomy is extensive decompression accompanied by foraminotomies and the essential undercutting of the facet joints [1,13,60], grounded in the premise that decompression within the achondroplastic spinal canal should occur both laterally and longitudinally. Furthermore, instability following extensive laminectomies may prove to be more incapacitating than the underlying primary pathology itself. The objective is to attain sufficient neural decompression rather than solely increasing the size of the osseous canal. The preferred method involves the stabilization of decompressed segments via transpedicular fixation [61,62]. Within the pediatric demographic, a wide laminectomy combined with fusion is preferred, as the immature spine exhibits increased susceptibility to instability and deformity. In the investigation conducted by Wang et al. [63], which examines spinal cord decompression and vertebral column resection in patients with achondroplasia, the mean correction rates for thoracolumbar kyphosis (TLK) and principal curves were determined to be 73 ± 15% and 87 ± 6%, respectively, meaning that TLK was corrected by 73°, and the principal curve was corrected by 87°. A total of five patients (71%) presented with neurological symptoms prior to surgery. Notably, all patients exhibited improvements in their neurological functions at the final follow-up assessment. Surgical complications were observed in four patients (57%), which included rod fractures (43%), neurological complications (28%), dural lacerations (14%), cerebrospinal fluid leaks (14%), and proximal junctional kyphosis (14%). The authors deduced that posterior vertebral column resection (p-VCR) can provide substantial correction of TLK and enhance neurological function through extensive laminectomies in individuals with achondroplasia. However, the incidence of surgical complications remains comparatively elevated. Consequently, it is regarded as a judicious surgical alternative for severe TLK in achondroplastic patients, particularly under the expertise of seasoned spinal surgeons, especially in cases involving markedly hypoplastic vertebrae at the apex [63]. In the research conducted by Tanaka et al. [64], all subjects experienced back pain, neurological deficits, and urinary disturbances prior to surgical intervention. Notably, all patients demonstrated neurological improvements postoperatively. The mean preoperative kyphotic angle was recorded at 117° (with a range of 103–126°). Postoperative measurements revealed an average kyphotic angle of 37° (ranging from 14° to 57°), culminating in a mean correction rate of 67%. All patients encountered postoperative complications, including rod breakage and/or surgical site infections. Findings suggest that the long-term outcomes of p-VCR are satisfactory for managing thoracolumbar kyphosis in achondroplastic patients. To execute this procedure safely, the implementation of spinal navigation and neuromonitoring is imperative when resecting non-anatomical fused vertebrae and ensuring the accurate placement of pedicle screws. Nevertheless, the occurrence of surgical complications, such as rod breakage and surgical site infection, may manifest at a significant frequency, thus emphasizing the necessity of informed consent when surgical intervention is warranted. In previously decompressed spines, recurrent stenosis (restenosis) may arise due to accelerated facet joint hypertrophy, excessive osseous overgrowth, and scarring. This hastened facet hypertrophy may indicate instability of the previously operated spine (especially in the absence of transpedicular stabilization) in achondroplastic individuals or represent an exaggerated response to normal motion attributable to the underlying genetic anomaly. Numerous studies have corroborated the efficacy of decompressive procedures for spinal canal stenosis in patients with achondroplasia; however, several investigations have indicated that reoperation due to restenosis is frequently necessitated [42,48,54,65]. In another study [66], among patients with achondroplasia treated for canal stenosis, the incidence of surgical intervention for spinal stenosis was reported at 22%, while the risk of revision surgery was found to be 38%, primarily attributed to pseudarthrosis, proximal junctional kyphosis (PJK), and recurrent neurological symptoms. Surgeons should contemplate spinal surgery as an integral component of patients’ overall management plan and should consider extensive decompression of the stenotic levels, along with long fusion employing interbody cages at the caudal level, in all patients to mitigate risks associated with revision surgery [66]. Despite these potential complications, patients with achondroplasia typically derive benefits from surgical interventions, as evidenced by a follow-up period of two years post-surgery [67]. In our case, despite complications that arose in the process, no new neurological deficits were permanent, and the whole procedure ultimately proved to be safe. An issue that had not been predicted, and therefore not planned for, was the effective disproportion of the patient’s limbs after spine correction was performed. According to Bednarczyk et al., limb-lengthening procedures are among the primary elements of multidirectional care, and they are the staple method of life quality improvement of these patients; this applies to both patients undergoing spinal deformity surgeries and those with no spine-related difficulties [57]. Therefore, through additional surgeries, this complication was resolved; however, it was impossible to lengthen the patient’s arm sufficiently, and so the procedure resulted in a deterioration of her quality of life. Her arm was lengthened by 6 cm, which, compared to the 13 cm she gained in height, did not suffice to make her capable of caring for herself. As Paley [68] specialized in extensive limb lengthening of patients with achondroplasia and hypochondroplasia, their results suggested that an average increase of 26 to 30 cm is possible. However, their research was focused on femur and tibia lengthening, which naturally differs from the results that are obtainable in the humerus. Per their method, a four-segment lengthening strategy, in any single bone segment, no more than 8 cm can be gained [68]. As for our patient, the lengthening process was limited by the aspect of her muscle and the fact that she did not opt to continue with more procedures after the first one did not meet her expectations. This indicates the need to account for such possible scenarios before the surgery and to plan it accordingly. Perhaps it should be considered whether or not the maximal achievable correction is necessary and if it should be reduced based on the expected height gain, as evaluated according to the current limb length. Imrie et al. compared the results of adolescent idiopathic correction between a group of patients with over 80% correction achieved and a group of patients with less than 40% correction achieved [69]. They found that while radiographically greater correction did show better results, there was little difference between what the patients reported about their own state, as reported on a questionnaire about the quality of life (SRS). With more similar cases, perhaps the process of estimating and planning the correction will be simplified once there are enough cases described to extract the most efficient way to design the intended outcome.

In a comprehensive multicenter investigation [70], data obtained from one thousand three hundred and seventy-four individuals diagnosed with achondroplasia were incorporated into the analysis. A total of four hundred and eight (29.7%) individuals had undergone at least one orthopedic surgical intervention throughout their lives, while 299 (21.8%) individuals experienced multiple surgical procedures. A proportion of 12.7% (n = 175) of the population underwent spinal surgery, with a mean age at initial surgery recorded as 22.4 ± 15.3 years. The median age at which surgery was performed was determined to be 16.7 years (range 0.1–67.4 years). Furthermore, 21.2% (n = 291) of individuals received surgical intervention on the lower extremities, with a mean age at first surgery of 9.9 ± 8.3 years and a median age of 8.2 years (range: 0.2–57.8 years). The predominant spinal procedure identified was decompression, with 152 patients undergoing a total of 271 laminectomy procedures, whereas the most frequently performed lower extremity procedure was osteotomy, with 200 patients undergoing a cumulative total of 434 procedures. Notably, fifty-eight (4.2%) patients underwent both spinal and lower extremity surgical interventions. Identified specific risk factors that augment the likelihood of orthopedic surgery include patients with hydrocephalus, necessitating shunt placement and exhibiting increased odds of undergoing spinal surgery; patients who underwent cervicomedullary decompression also demonstrated elevated odds of spinal surgery, and the occurrence of lower extremity surgery was associated with heightened odds of subsequent spinal surgery. The authors concluded that orthopedic surgery is a prevalent occurrence among individuals with achondroplasia, with 29.7% of patients having undergone at least one orthopedic procedure. Spine surgery (12.7%) was found to be less frequent and typically occurred at a later stage compared to lower extremity surgery (21.2%), while no information about upper extremity surgery was provided [70]. The presence of cervicomedullary decompression and hydrocephalus requiring shunt placement was correlated with an increased risk for spinal surgical interventions. The findings from the CLARITY study, which represents the most extensive natural history investigation of achondroplasia, are anticipated to assist clinicians in providing informed guidance to patients and their families regarding orthopedic surgical options. Another salient concern in achondroplasia, which was not observed in our patient but merits particular scrutiny and discourse, pertains to the abnormal anatomy of the craniovertebral junction and the associated risk of cervicomedullary compression. Cervicomedullary compression arises from a diminished diameter of the foramen magnum in both the sagittal and coronal planes, occasionally exceeding five standard deviations below normative values [4,27,71]. The management of cervicomedullary compression necessitates prompt and assertive intervention. Empirical evidence indicates that this condition is progressive and potentially lethal, as it heightens the likelihood of sudden death resulting from central respiratory failure [16,27,32,33,34]. This pathology is increasingly acknowledged as a contributing factor to respiratory and neurological anomalies in pediatric patients diagnosed with achondroplasia [34,35,36].

## 5. Conclusions

Skeletal dysplasia comprises a heterogeneous group of disorders that require a multidisciplinary approach and integrated treatment strategies for effective management. Although neurosurgical procedures in children involve inherent risks, these can be significantly reduced through appropriate clinical expertise, surgical experience, and well-trained support teams. Spinal canal stenosis occurs more frequently than cervicomedullary compression, and modified laminectomy techniques are associated with better outcomes than traditional methods. Craniovertebral decompression may be a life-saving intervention and can improve the natural course of achondroplasia by supporting neurological and developmental progress in young patients. While patients with achondroplasia have a general anatomical predisposition to hydrocephalus, most tolerate its manifestations relatively well; therefore, in selected cases, conservative management may be appropriate. Despite the rarity of achondroplasia, the high prevalence of central nervous system involvement offers meaningful opportunities to significantly improve long-term outcomes.

Posterior vertebral column resection (VCR) remains one of the most effective surgical techniques for managing thoracolumbar kyphosis (TLK) in patients with achondroplasia, particularly following extensive laminectomies where neurological decompression is required. However, given the high incidence of surgical complications associated with this procedure, VCR should be reserved as a surgical option for severe TLK in achondroplasia patients and performed exclusively by experienced spinal surgeons, particularly in cases with markedly hypoplastic apical vertebrae. As of now, there is no scale that can facilitate such qualifications for VCR. The use of multiple-rod constructs with solid fusion is recommended to enhance stability and reduce revision rates. Spinal navigation and intraoperative neuromonitoring are valuable adjuncts for safe vertebral resection and accurate pedicle screw placement. Although advances in technique have reduced complication rates, transpedicular screw-related injuries remain a major challenge, requiring thorough anatomical knowledge, meticulous techniques, and careful preparation. Surgeons must also avoid overcorrection to preserve functional body proportions, as excessive correction may impair daily functioning and necessitate further procedures.

## Data Availability

No new data were created or analyzed in this study.
